# Formulation and Characterization of Emulgel-Based Jelly Candy: A Preliminary Study on Nutraceutical Delivery

**DOI:** 10.3390/gels9060466

**Published:** 2023-06-06

**Authors:** Somali Dhal, Anupam Pal, Anna Gramza-Michalowska, Doman Kim, Biswaranjan Mohanty, Sai S. Sagiri, Kunal Pal

**Affiliations:** 1 Department of Biotechnology and Medical Engineering, National Institute of Technology Rourkela, Rourkela 769008, India; somali.dhal12@gmail.com; 2 Department of Pharmaceutics, Institute of Pharmacy and Technology, Salipur, Cuttack 754202, India; anupampal003@gmail.com; 3 Department of Gastronomy Science and Functional Foods, Faculty of Food Science and Nutrition, Poznań University of Life Sciences, Wojska Polskiego 31, 60-624 Poznań, Poland; anna.gramza@up.poznan.pl; 4 Graduate School of International Agricultural Technology, Seoul National University, Gangwon-do, Seoul 25354, Republic of Korea; kimdm@snu.ac.kr; 5 Agro-Nanotechnology and Advanced Materials Research Center, Department of Food Science, Agricultural Research Organization, The Volcani Institute, Rishon Lezion 7505101, Israel; biotech.satishsai@gmail.com

**Keywords:** curcumin, riboflavin, emulgel, jelly candy, sucrose, pectin, olive oil, nutraceutical

## Abstract

The development of consumer-friendly nutraceutical dosage forms is highly important for greater acceptance. In this work, such dosage forms were prepared based on structured emulsions (emulgels), where the olive oil phase was filled within the pectin-based jelly candy. The emulgel-based candies were designed as bi-modal carriers, where oil-soluble curcumin and water-soluble riboflavin were incorporated as the model nutraceuticals. Initially, emulsions were prepared by homogenizing varied concentrations (10% to 30% (*w*/*w*)) of olive oil in a 5% (*w*/*w*) pectin solution that contained sucrose and citric acid. Herein, pectin acted as a structuring agent-cum-stabilizer. Physico–chemical properties of the developed formulations were thoroughly analyzed. These studies revealed that olive oil interferes with the formation of polymer networks of pectin and the crystallization properties of sugar in candies. This was confirmed by performing FTIR spectroscopy and DSC studies. In vitro disintegration studies showed an insignificant difference in the disintegration behavior of candies, although olive oil concentration was varied. Riboflavin and curcumin were then incorporated into the jelly candy formulations to analyze whether the developed formulations could deliver both hydrophilic and hydrophobic nutraceutical agents. We found that the developed jelly candy formulations were capable of delivering both types of nutraceutical agents. The outcome of the present study may open new directions for designing and developing oral nutraceutical dosage forms.

## 1. Introduction

Oral administration of pharmaceutical dosage forms has been considered the most versatile patient compliance administration method for a long time. More than 60% of bioactive agents are orally administrated [1]. In comparison to other modes of delivery, the oral route lowers the risk of disease transmission, is cost-effective, and increases patient compliance. The disadvantages of oral administration are less absorption, poor solubility, poor permeability, and fast degradation in the gastrointestinal tract. Therefore, numerous strategies have been extensively researched to circumvent the drawbacks of therapeutic drug delivery via the oral route. A novel candy-based drug delivery system has been proposed for this approach that minimizes the disadvantages [2]. Due to their higher acceptability among consumers, these candies are a promising formulation to fortify with different bioactive compounds such as antioxidants, nutraceuticals, etc. Such formulations are expected to effectively deliver bioactive ingredients for human health and benefit wide demography, ranging from kids to adults [3,4,5].

**Scheme 1:**
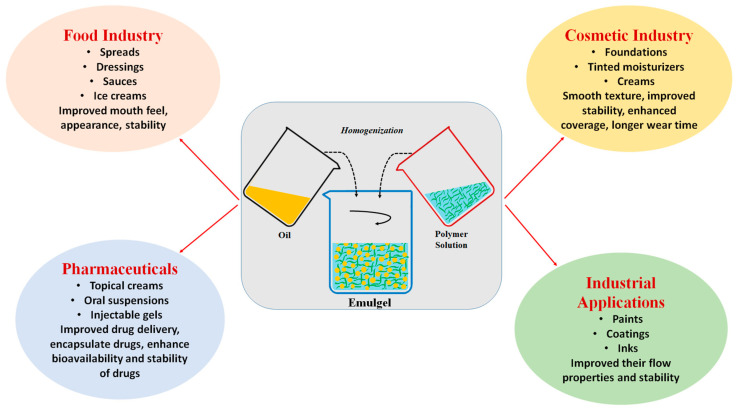
Emulsification of gels and their application in various industries.

Gels are formed by a three-dimensional network of interconnected particles or molecules that trap liquid within their structure [6,7]. The liquid can be water, oil, or any other solvent, and the particles or molecules that form the network can be polymers, proteins, or other organic or inorganic materials. The materials can interact through chemical or physical processes to create a stable gel structure. Depending on the nature of the materials and the gelation process, various properties, such as texture, stiffness, and water-holding capacity, can be modulated. Hydrogels and organogels are the most commonly used gels in the pharmaceutical, food, and cosmetic industries. However, the limitations associated with them constrain their application for the delivery of specific hydrophobic and/or hydrophilic bioactive compounds. According to Farzami et al. (2019), the hydrophilic nature of hydrogels limits their applicability in delivering hydrophobic molecules [8]. In such scenarios, the emulsification of gels can be an alternative for similar applications. Fabricating emulsion gels/emulgels can be performed by embedding an emulsion into either a gel phase or a pre-gel polymer solution, followed by gelation. The gel network becomes tightly packed during the setting process, forming a network from flocculated droplets, resulting in a firmer and more stable gel. The overall process of emulsification of gels and their probable applications in various sectors was shown in Scheme 1. Eventually, the formulated hybrid systems can provide the advantages of emulsions and hydrogels, i.e., the ability to solubilize hydrophobic and hydrophilic components and good thermodynamic stability. The literature has reported that emulgels can be used as fat replacers in food products, and they can also be used as delivery systems to encapsulate and release bioactive compounds [9]. Emulsion gels combined with animal fat have been suggested to make lower-calorie meat products without sacrificing flavor. By altering sodium mobility and binding behavior, emulsion gels help manage sodium availability and perception, allowing for lower salt content in meat products [10]. Several studies have reported using emulsion gels to deliver bioactive compounds, including curcumin, β-carotene, α-tocopherol, and vitamin A [11]. The use of emulgels as delivery systems helps to improve the controlled release of bioactive ingredients during oral processing and gastric digestion [12,13].

In the confectionery food segment, jelly candies account for nearly 50% of the market, becoming a popular health supplement for millions [14]. Most children prefer soft confectionery foods such as pastilles, edible jelly, and gummy candies because they are easier to swallow and have a pleasant taste [15]. These normally consist of a large amount of sugar with a certain amount of gelling agents, such as pectin, gelatin, agar, etc. [1]. Pectin, a plant-derived anionic hydrocolloid, is one of the most commonly employed gelling agents [16]. It is also a commonly used biopolymer that is used in several scientific fields due to its low cost, availability, safety, and functionality [17]. Pectin is a polysaccharide found naturally in a variety of plants, and the major constituent of pectin is galacturonic acid [18]. The three-dimensional water-insoluble network structure of pectin can entrap and immobilize various biologically-active molecules when it undergoes cross-linking. Pectin gel has unique properties, making it a perfect material for designing nutrient and medication delivery systems [19]. Nevertheless, there is little knowledge on adding emulsion to food products with a gel-like structure, such as jelly candies [3]. The emulgel matrix is a complex colloidal system obtained by replacing the hydro phase of the gel (partially or totally) with an emulsion [20]. Thus, emulgel-based jelly candies are suitable for delivering lipophilic and hydrophilic bioactive agents by emulsifying the candy formulations [21]. In this regard, we used olive oil, which acts as a lipid phase. Olive oil is the most preferred edible oil due to monounsaturated fatty acid, i.e., oleic acid, which helps decrease the risk of oxidative deterioration [22]. The incorporation of olive oil in the jelly candy matrix can improve the properties of the candy matrices as prospective delivery systems [23]. Due to its organoleptic qualities in association with positive health effects, olive oil has become more popular in recent years [24]. 

Our preliminary literature review exposed that an investigation into emulgel-based jelly candy formulations has yet to be carried out [25,26,27]. Therefore, in this present study, we primarily concentrated on the development and characterization of emulsified jelly candy formulations of pectin and olive oil, wherein the composition of the biphasic system was varied. The formulated jelly candies were characterized to understand the physicochemical, thermal, mechanical, and drug release properties. Such characterizations were performed by conducting a transparency study, pH measurement, disintegration test, color analysis, FTIR spectroscopy, impedance analysis, and mechanical testing. The thermal behavior of the candy formulations was studied through differential scanning calorimetry (DSC). In order to test our hypothesis that the emulgel candies can deliver both hydrophilic and hydrophobic agents, we incorporated riboflavin and curcumin into the formulations and studied their release properties. Subsequently, their release profile was also analyzed.

## 2. Results and Discussion

### 2.1. Preparation of Candy

**Figure 1:**
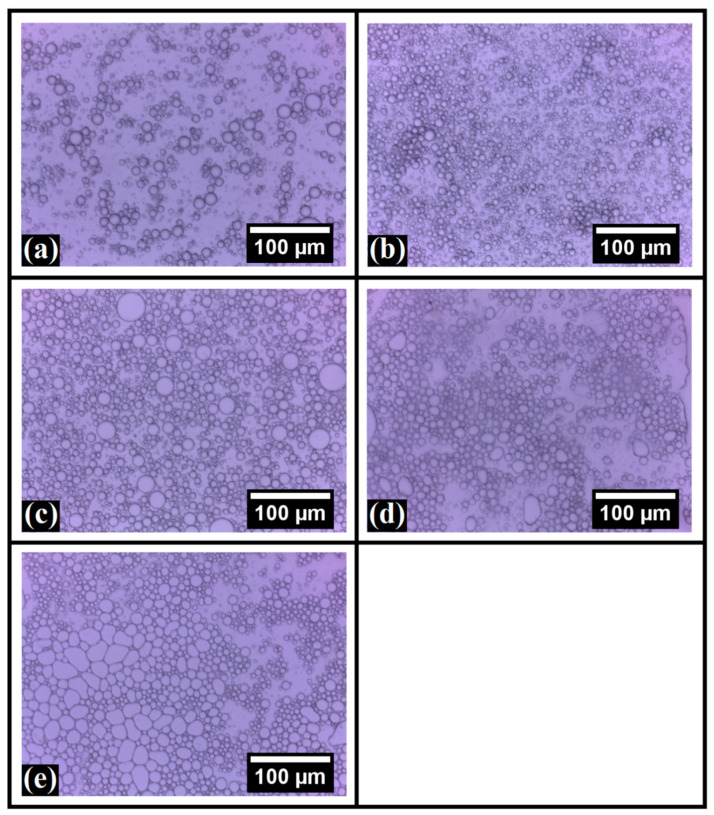
Micrographs of emulsions of varying olive oil concentration (**a**) 10%, (**b**) 15%, (**c**) 20%, (**d**) 25%, and (**e**) 30% *w*/*w*.

Sucrose solution, citric acid, pectin solution, and olive oil were used to develop the emulgel candies. The control candy (AP00) was prepared by mixing SCS with the structuring agent (pectin solution). Pectin is a polysaccharide that requires certain suitable conditions to form a gel. Previous studies have reported that pectin molecules require a low molecular co-solute that acts as a dehydrating agent (e.g., sucrose) to initiate gelation [28]. The literature has also stated that citric acid promotes the gelation of anionic polymers, including pectin [29]. The addition of citric acid lowers the pH and induces pectin gel formation. When the pH of the solution is reduced, the ionization of the carboxylate groups is suppressed. This results in the conversion of highly hydrated carboxylate groups of pectin into mildly hydrated carboxylic acid groups. Hence, the pectin molecules no longer reject each other along the polymer length due to losing their charge. Due to this reason, the pectin polymer chains associate themselves along a portion of their length to form a gel. There were also reports that the -COOH chain of the pectin molecule provides stability to the gel matrix via steric and electrostatic interactions [22]. A previous study reported that pectin acts as an anchor for the emulsification of oil droplets [30]. The insoluble pectin gelator network facilitated the entrapment of olive oil and formed structured O/W emulsions, as shown in Figure 1. All the formulations showed multiple-sized droplets, irrespective of the olive oil volume. The size and droplet density seems to have increased along with the olive oil content. Oil droplets in AP30 (the formulation containing 30% (*v*/*v*) olive oil) were densely packed and deformed (Figure 1e). This indicates that adding olive oil has significantly altered the structural integrity of the candy formulations. 

**Figure 2:**
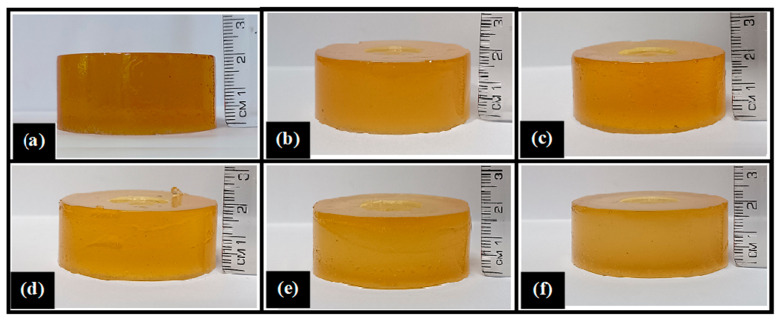
Emulgel-based candy formulations. (**a**) AP00, (**b**) AP10, (**c**) AP15, (**d**) AP20, (**e**) AP25, and (**f**) AP30.

**Figure 3:**
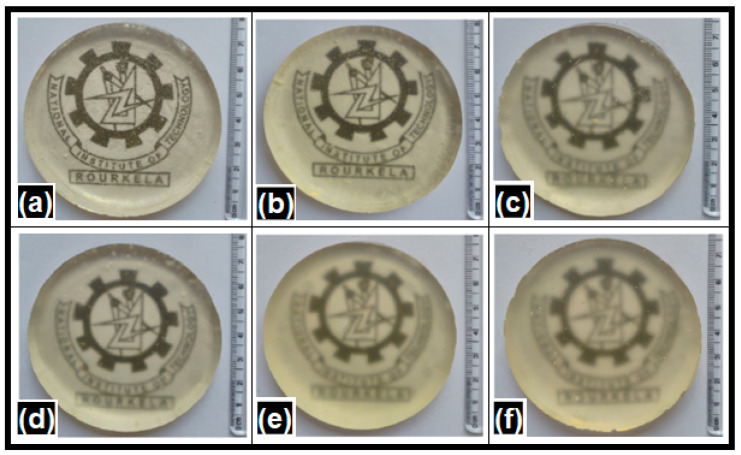
Transparency study of emulgel-based candy formulations. (**a**) AP00, (**b**) AP10, (**c**) AP15, (**d**) AP20, (**e**) AP25, and (**f**) AP30.

Preliminary investigation showed that the concentration of pectin significantly affects the preparation of jelly candies. Softer candies were formed when pectin concentration was up to 30% (*w*/*w*) in the formulation. Thus, the candy with 30% PS was chosen as the control formulation (AP00). AP00 was a brownish solid, non-sticky, elastic, and thermally irreversible. Adding citric acid to the sucrose solution turned the formulations yellowish–brown in color (Figure 2a). The inclusion of olive oil resulted in color differences when observed through the naked eye. All the candies were transparent (Figure 3). AP00 was a highly transparent candy, and the transparency started decreasing with the increase in olive oil concentration in the candy formulations. Similar observations were reported by Pan et al. (2022), who investigated the droplet distribution in xanthan gum-based soft candies. They observed that as the concentration of the gum increases, oil droplets become more crowded, creating a more uniform distribution in the candy formulations [31].

### 2.2. Colorimetry Analysis

**Figure 4:**
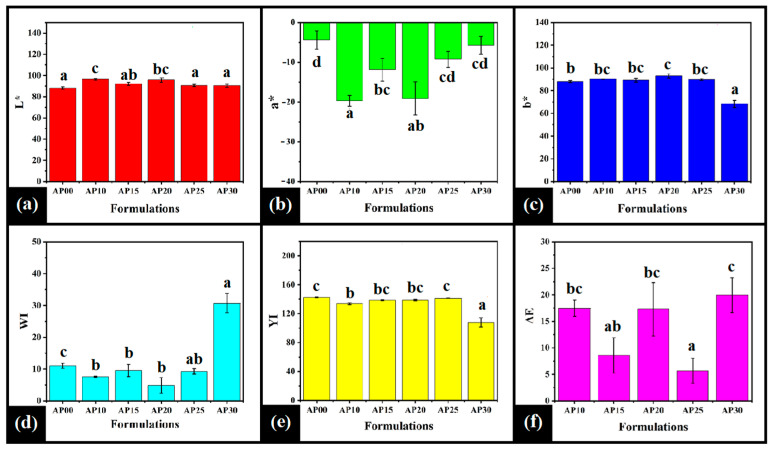
Colorimetry analysis of emulgel-based candy formulations. (**a**) L*, (**b**) a*, (**c**) b*, (**d**) WI, (**e**) YI, and (**f**) ΔE. Those not sharing the same letter(s) in each chart significantly differ, as per the Tukey HSD test with *p* < 0.05.

The color parameter of any food material is a significant index in terms of overall appearance, quality, genuineness, and consumer acceptance. The effect of olive oil addition based on Commission Internationale de l’Eclairage (CIE) color measuring coordinates, i.e., L* (black to white), a* (green to red), b* (blue to yellow), WI, YI, and ΔE are presented in Figure 4. AP00 appeared to be clearer and more transparent. The addition of olive oil into the formulation affected the color parameters of the candy.

The average lightness values (L*) of the candy formulations ranged from 88 to 90 (Figure 4a), indicating the luminous property of the formulations [32]. When olive oil was added, there was no discernible change in the appearance of the food gel. Hence, the high L* values are acceptable [33]. AP00 showed the lowest L* value among all the formulations. However, the incorporation of olive oil in the formulation increased L* values. Previous study results showed that the values of L* for olive oil range between 91.5 and 97.2 [34]. Compared to the control, AP10 and AP20 had significantly higher L* values (*p* = 0.001). The rest of the formulations were similar to that of the control (*p* > 0.05). Overall, olive oil showed an insignificant effect on the L* values of the formulations.

The average a* values of the formulations lie within a negative value region between −19 and −4 (Figure 4b). The previous literature reported that negative values of a* indicate the development of a greenish color [34]. AP00, which did not contain olive oil, had the lowest a* value. The subsequent incorporation of olive oil resulted in a significant increase in the a* value in AP10 (*p* = 0.001), AP15 (*p* = 0.042), and AP20 (*p* = 0.001) compared to the control. However, a further increase in the olive oil content lowered the a* values in AP25 and AP30, wherein the a* values were similar to AP00. Among the olive oil-containing formulations, AP10 and AP20 had the highest values (*p* > 0.05). Therefore, a* values suggest that the inclusion of olive oil played a significant role in developing greenness in the formulations. However, a* values did not follow any trend with the increased olive oil concentration (Figure 4b).

According to the CEILAB color coordinates, positive b* values indicate the yellowish tone in the formulations. All the formulations had a b* value ranging from 68 to 93 (Figure 4c). The b* value for the control sample (AP00) was statistically similar compared to the olive oil-containing formulations except for AP20 (*p* = 0.027) and AP30 (*p* = 0.001). The addition of olive oil increased the yellow tone in the formulation till AP25. Subsequently, a sharp decrease in the b* value was noticed in AP30, which showed the highest standard deviation of b* values among all the formulations.

The WI is the intensity of light reflected in all directions by an ideal reflecting surface that neither absorbs nor transmits light. The WI of the AP00 had a value of 11.05 ± 0.78. The addition of olive oil lowered the WI values significantly in all the formulations except for AP30 (Figure 4d). The statistical pattern of WI followed a similar trend as observed with the b* values, where AP30 significantly differed from the others (*p* < 0.05). The YI of the formulations also followed the trend as that of b* values (Figure 4e). Among all, the control formulation showed the highest YI value of 142.61 ± 0.53. The addition of olive oil resulted in lower YI values. Among the olive oil-based formulations, AP25 showed the highest YI value, while AP30 showed the lowest YI value. The YI value of the rest of the formulations was found to be similar to that of the control sample.

The total color difference, ΔE, was calculated using Equation (3) by considering AP00 as the standard. ΔE values of the candy formulations ranged between 6 and 20 (Figure 4f). The ΔE of all the candy formulations was greater than five, indicating that the human eye can perceive the color differences in the formulations with respect to the control sample (Figure 2) [35].

### 2.3. pH Analysis

The pH of candy formulations plays a critical role in their stability. Pectin exhibits a higher degree of polymerization at a lower pH value [36]. This is due to the strong intermolecular interaction among the pectin chains, thereby increasing the mechanical stability of the pectin formulation. The pH of the developed candy formulations ranges between 3.03 and 3.45 (Figure S1). The low pH of the candy formulations can be explained by the presence of citric acid in the formulations. The pH value of the emulgel candies showed statistically no difference (*p* > 0.05; Figure S1) compared to the control formulation. An increase in the concentration of olive oil did not affect the pH of the formulations. 

### 2.4. FTIR Spectroscopy

**Figure 5:**
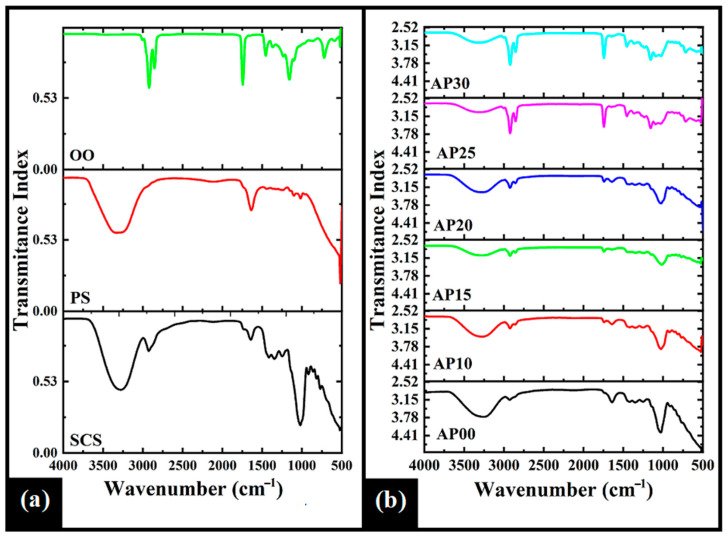
FTIR spectra of (**a**) raw materials and (**b**) candy formulations. OO—Olive oil, PS—Pectin Solution, SCS—Sucrose Citric Acid Solution.

FTIR spectroscopy is a versatile tool that helps to identify the structural arrangement and chemical interactions between the formulated candy components by observing the corresponding absorption peaks of functional groups (Figure 5; Table S1). FTIR spectra of SCS showed absorption peaks at 3279, 2926, 1642, 1414, 1345, and 1022 cm^−1^. A broad, strong absorption peak at 3279 cm^−1^ was due to the O-H stretching vibration of the water molecules in the SCS solution and the alcohol groups associated with the sucrose molecule [37]. The shoulder peak obtained at 2926 cm^−1^ can be attributed to the asymmetric C-H stretching of aliphatic groups of the sucrose molecule [38]. At 1642 cm^−1^, a weak absorption band was observed, which can be related to the deformation of water molecules embedded within the sucrose matrix [39]. Small and weak peaks were also obtained at 1414 and 1345 cm^−1^ in the FTIR spectrum of SCS. The peak at 1414 cm^−1^ arose due to the twisting of the water molecules after bonding with sucrose molecules in the SCS solution [40]. The peak at 1345 cm^−1^ can be assigned to the symmetric scissoring of the C-OH groups in citric acid [41], whereas the strong peak at 1022 cm^−1^ can be attributed to C–C bonds stretching in sucrose [41].

The FTIR spectrum of PS showed characteristic peaks at 3328, 1636, 1102, and 1018 cm^−1^. The strong and broad peak at 3328 cm^−1^ refers to the O-H vibration of the hydroxyl group related to intra- and inter-molecular H-bonds of the galacturonic acid present in pectin [42]. Further, the medium intense peak at 1636 cm^−1^ indicates the C=O stretching vibration of the carboxylate ion (COO^−^). The weaker symmetric COO^−^ stretching in pectin samples can be identified by observing the moderately intense absorption patterns in the fingerprint region of 1300 to 800 cm^−1^ [43]. Additionally, the absorption band at the wavenumber 1018 cm^−1^ suggests the presence of natural sugars and galactose [44]. In PS spectra, a strong and broad peak was obtained at 3326 cm^−1^, indicating the stretching of hydroxyl groups in the glucopyranose ring. A small and weak band at 1636 cm^−1^ was obtained due to the presence of carboxylate anion (COO^−^) [45].

FTIR-spectrum pure olive oil is shown in Figure 5a. Major peaks corresponding to the fatty acid composition of olive oil are summarized here. The conjugative sharp bands at 2924 cm^−1^ and 2861 cm^−1^ are due to the antisymmetric and symmetric stretching vibrations of aliphatic C-H in -CH_2_ and terminal -CH_3_ groups, respectively [46]. In addition, the sharp peak at 1743 cm^−1^ arose because of the C=O (ester peak) stretching vibration of the carbonyl group of triacylglycerols [47]. The fingerprint region (1455–721 cm^−1^) of the spectrum shows the bending and rocking vibration of aliphatic groups. CH_2_ scissoring was confirmed due to the presence of the peak at 1455 cm^−1^ [48]. The characteristic peak at 1366 cm^−1^ is associated with the asymmetric H-C-H bending of the glycerol group’s O-CH_2_ corresponding to mono, di, and triglycerides [49]. The band at 1158 cm^−1^ is assigned to the C-O stretching attributed to the tertiary alcohols [50]. Finally, the prominent peak observed at 721 cm^−1^ may be attributed to the superimposition of the alkyl skeletal rocking oscillation and out-of-plane vibration (-CH wagging) of cis-di-substituted olefins [47].

The FTIR spectra of the emulgel-based candy formulations are represented in Figure 5b. Similar spectral features were seen in AP00 (control candy formulation) and the sucrose citric acid solution. A broad, strong band at 3256 cm^−1^ can be associated with stretching vibrations of -OH group due to intermolecular H-bonding among the -OH groups of pectin and the SCS solution (Santos et al., 2020). The developed candy formulations have shown nearly all the absorption peaks as presented in the raw materials. Interestingly, the incorporation of olive oil resulted in a gradual decrease in the peak intensity at 3277 cm^−1^ from AP00-AP30, except AP20. This indicated a decrease in the intermolecular hydrogen bonding with the increased olive oil content. The appearance of absorption peaks at 2926 cm^−1^ in AP00 might be because of the formation of physical bonds between the components [51]. In AP25 and AP30, the absorption peak trend was similar to that of olive oil. This might be due to the saturation of the emulsion with the oil phase [52,53]. This can be correlated with the candy microstructures (Figure 1d,e), where insignificant phase separation was noticed.

### 2.5. DSC Analysis

**Figure 6:**
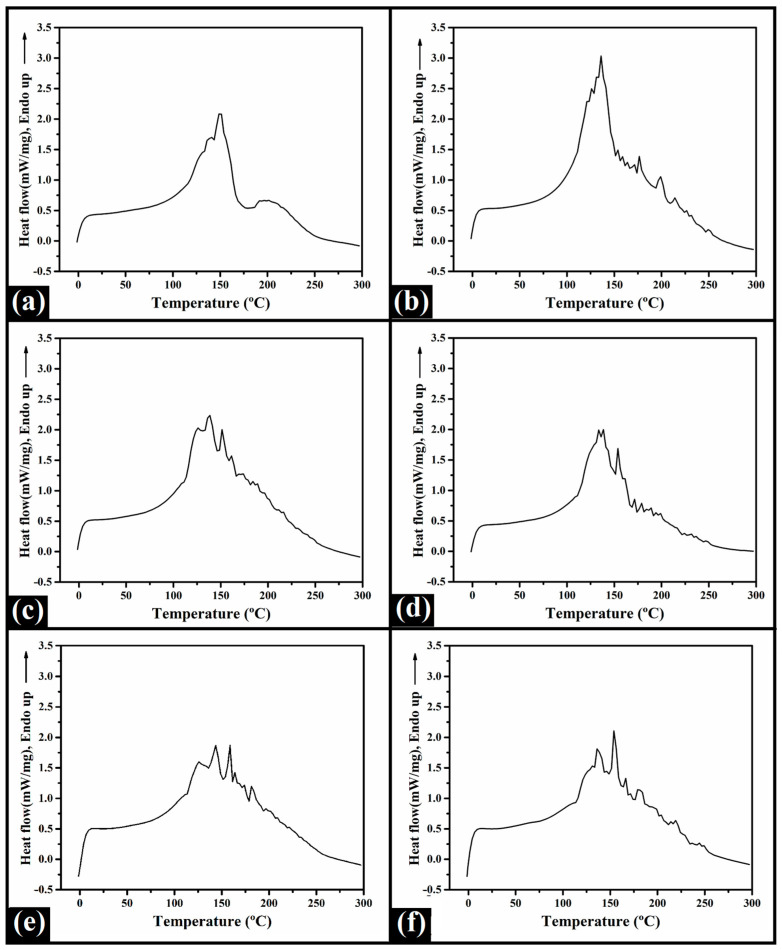
DSC thermograms of emulgel-based candy formulations. (**a**) AP00, (**b**) AP10, (**c**) AP15, (**d**) AP20, (**e**) AP25, and (**f**) AP30.

DSC is the most common method for evaluating the thermal properties of food products. The thermal-dependent process is always associated with the absorption or emission of energy in the form of heat. Thus, the area under the endothermic peaks quantitatively provides the amount of heat absorbed during the melting. It also presents the thermal behavior of the ingredients present in the samples. In other words, the thermal events related to the raw materials used to develop the formulations are also presented. In our case, the primary constituent of the candy formulations is SCS. Sugars (e.g., sucrose, fructose, and glucose) play a significant role in determining the stability of polysaccharide gel-based food products. In sugar-containing polysaccharide gels, the sugars can affect the interactions between the polysaccharide chains and water molecules [54]. This results in differential hydration of polysaccharides in the presence of sugars. The endothermic curve of candy formulations showed two types of peaks, i.e., broad peaks and sharp peaks (Figure 6). In AP00, the initiation of the thermal event (~40 °C) was due to the evaporation of loosely bound water molecules. After 50 °C, the heat flow increased, and a small hump appeared at ~128 °C (peak 1), which can be associated with the evaporation of entrapped water molecules in the jelly candy formulations. A further temperature increase led to another broad peak at ~138 °C (peak 2). This broad peak might have arisen due to the evaporation of the bound moisture in the pectin molecules [55]. Subsequently, another sharp endothermic peak (peak 3) was observed at ~148 °C. This sharp peak is due to the disruption of the crystalline structure of the sucrose molecules [56]. It has been reported that the melting temperature (T_m_) of sucrose is 150 °C at a thermal scanning rate of 10 °C/min [57]. The minor decrement in the T_m_ can be explained by the presence of pectin within the candy formulations [58].

Candy formulations (AP10 to AP30) showed a shift in the endothermic peak as compared to the control formulation (AP00). This shifting of the peaks can be related to the addition of olive oil to the formulations. Except for AP10, other formulations showed a great variation in peak intensity (Figure 6c–f). AP10 showed the highest peak among all the formulations. The high intensity of the sharp peaks suggests that sucrose molecules had crystallized better than in the other candy formulations [59]. Interestingly a peak was seen in the range of 151 °C and 158 °C. This particular peak intensity gradually increased with the olive oil concentration in the candy formulations and can be correlated with the FTIR analysis, where a decrement of intermolecular hydrogen bonding was observed with the addition of olive oil into the candy formulations.

### 2.6. Mechanical Analysis

**Figure 7:**
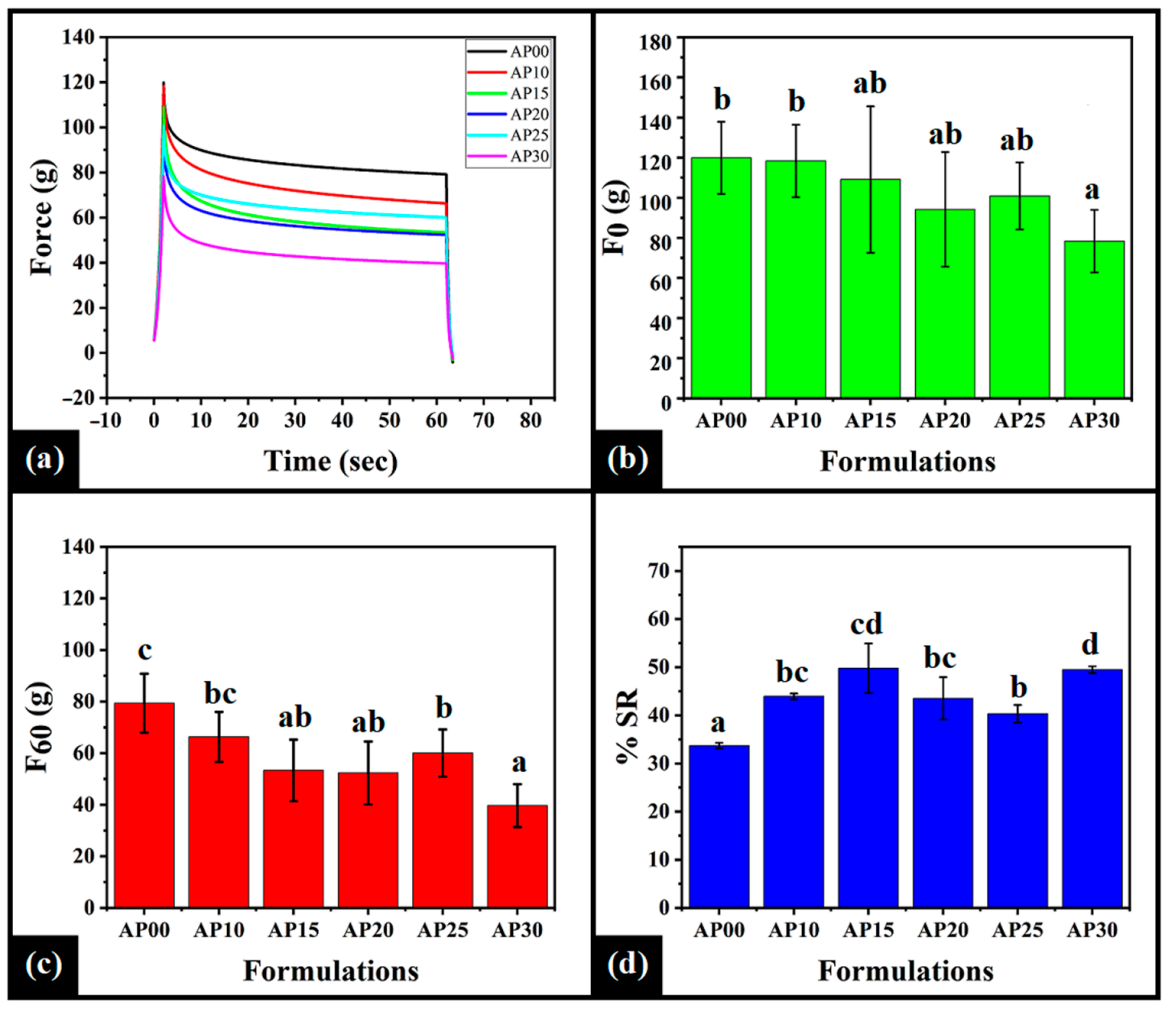
Stress relaxation profiles of the emulgel-based candy formulations: (**a**) stress relaxation profile, (**b**) F_0_ profile, (**c**) F_60_ profile, (**d**) %SR profile. Those not sharing the same letter(s) for each chart significantly differ by Tukey HSD test with *p* < 0.05.

The texture of candy is determined by the arrangement and interaction of different ingredients at both the microscopic and macroscopic levels, and it profoundly affects the acceptance by the customer [31]. The SR profiles of candy formulations were analyzed to determine the mechanical properties of the formulations when stress was applied (Figure 7). This study provides information about the viscoelastic nature of the formulations. The maximum force attained during the compression process is defined as “F_0_”, indicating the firmness of the formulations. When the force reached a maximum, the testing probe maintained the same position for 60 s. As time progressed, applied force decreased exponentially due to the extent of material relaxation, which depended on the composition of formulations. The force experienced by the load cell after 60 s is termed the “residual force” (F_60_). It was observed that the AP00 had a higher firmness than the rest of the formulations. The higher firmness of AP00 can be attributed to the maximum intermolecular interactions, as described in the FTIR analysis. The incorporation of olive oil into the candy formulations resulted in the decrement of the F_0_ values in a concentration-dependent manner. However, the F_0_ value of AP30 was significantly lower than AP00 (*p* < 0.05). Among the olive oil-containing formulations, the F_0_ value of AP10 and AP30 was statistically different (*p* = 0.001). The F_60_ value of the control (AP00) and emulgel-based formulations (AP10 to AP30) overall followed a similar trend as the F_0_ values. 

The percentage relaxation of stress (%SR) in the formulations was evaluated using Equation (4) [60]. Figure 7d confirms the higher impact of olive oil on stress relaxation, that is, the mechanical properties of the formulations. The %SR values of the candy formulations ranged between 33 and 50%, suggesting that the formulations predominantly consisted of elastic components [61]. In general, there was an increase in the %SR of the formulations as the olive oil content was increased until AP15. Thereafter, the %SR decreased until AP25. Interestingly, the %SR was increased in AP30, which was similar to that of AP15 (*p* = 0.984). It can be observed that the incorporation of olive oil in the candy formulations increased the %SR as compared to AP00 (*p* < 0.05) and, hence, the viscous component in the candy formulations. Interestingly, AP15 showed a higher standard deviation for %SR value than the rest of the formulations, which may be explained by the higher crystalline nature of the sucrose molecules [62,63]. 

### 2.7. In Vitro Disintegration Test

**Figure 8:**
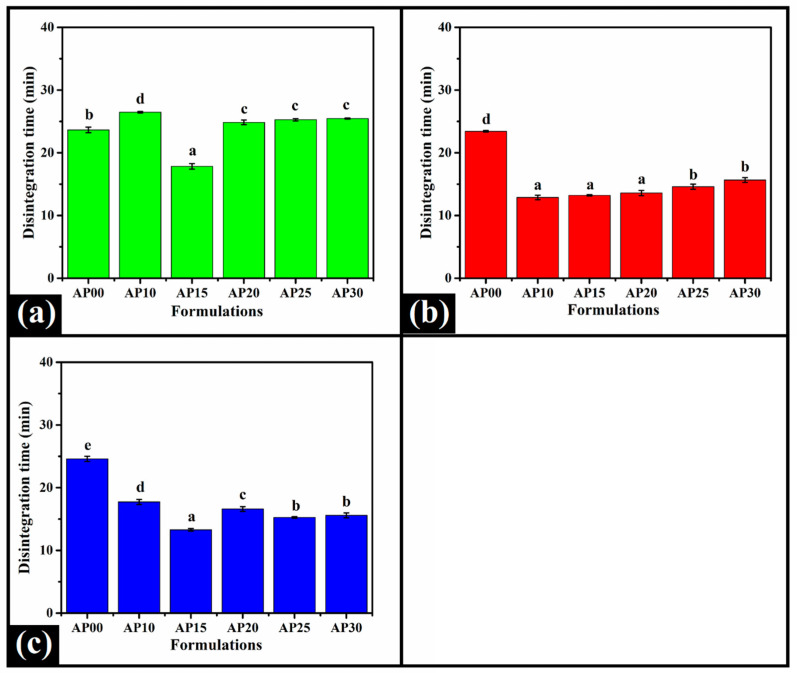
Disintegration times of the candy formulations at (**a**) pH 1.2, (**b**) pH 5.8, and (**c**) pH 7.2. Columns not sharing the same alphabet means they are statistically different values by Tukey HSD test with *p* < 0.05.

The in vitro disintegration times of the candy formulations in different pH media are shown in Figure 8. In stimulated gastric pH (pH 1.2), the disintegration time (DT) of AP00 was 23 ± 0.45 min. The incorporation of olive oil increased the DT of the candy formulations, except AP15. Among the olive oil-containing formulations, AP10 showed the highest DT, followed by AP20, AP25, and AP30, which showed a similar DT (*p* > 0.05). The DT of AP15 was the lowest among all the formulations (*p* = 0.001). 

When the pH of the medium was increased to 5.8 (simulated duodenal fluid), a dramatic decrease in the DT was noticed in the olive oil-containing candy formulations as compared to the control (AP00). The DT of AP00 was at pH 5.8, and pH 1.2 was similar. Such a result was expected because the -COOH groups in pectin and the citric acid become ionized at a higher pH. Further, an increase in the pH of the disintegrating medium to 7.2 (simulated intestinal fluid; Figure 8c) and a decrease in the DT of the candy formulations were observed as compared to AP00. Among all the formulations, a significant reduction in DT was observed in AP15, which was statistically significant against other formulations (*p* < 0.05). In short, the DT of AP00 at all three pHs was similarly valued (*p* > 0.05). However, the incorporation of olive oil considerably tailored the DT of the candy formulations, which can be explained by the presence of carboxylic acid groups in the fatty acids of olive oil.

### 2.8. Drug Release Study

**Figure 9:**
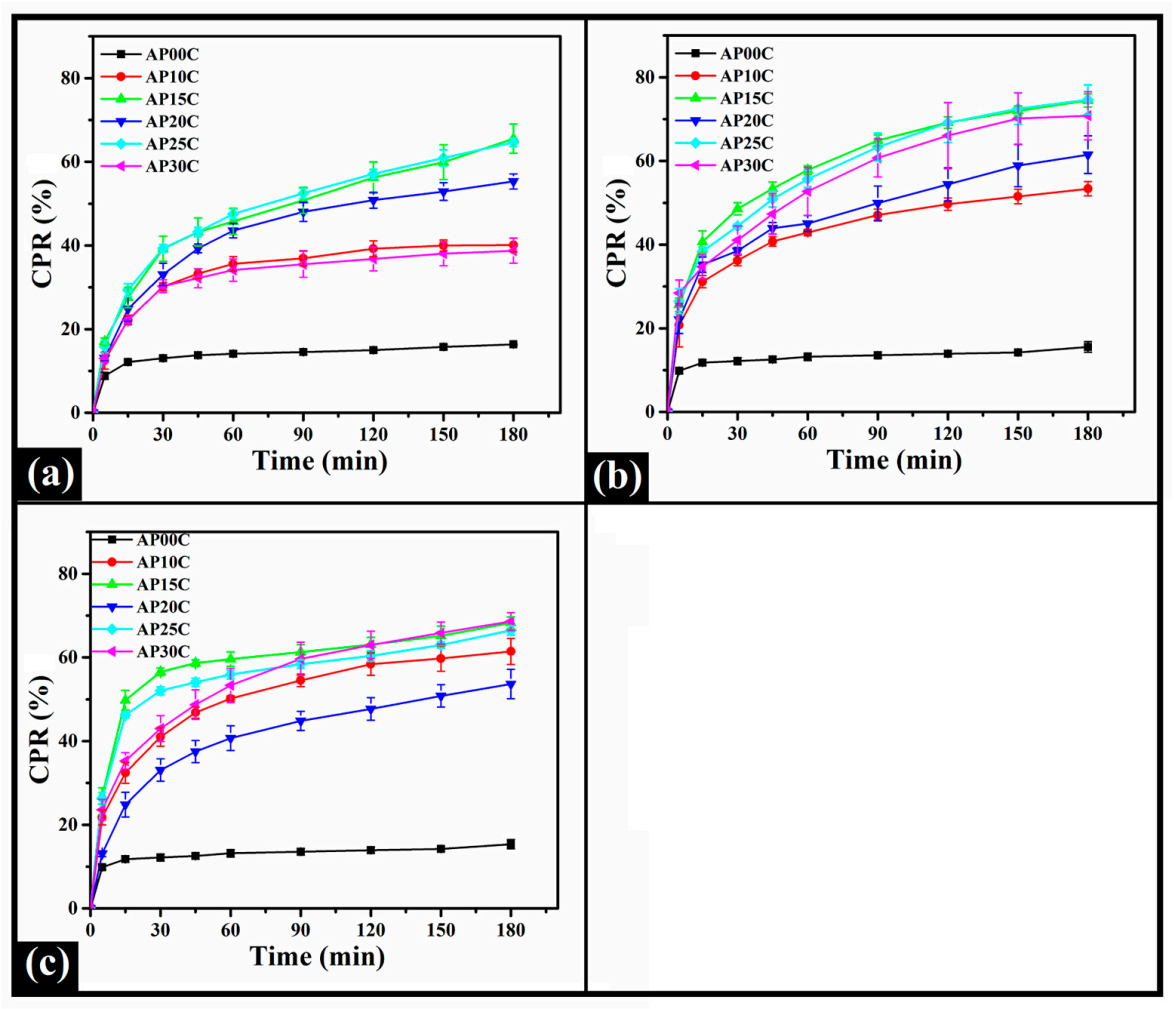
Dissolution profile of the candy formulations containing curcumin (**a**) CPDR (pH 1.2), (**b**) CPDR (pH 5.8), and (**c**) CPDR (pH 7.2).

The in vitro curcumin and riboflavin release from the candy formulations were evaluated in simulated gastric pH (1.2), simulated duodenal pH (5.8), and simulated intestinal fluid (7.2), separately. For curcumin, the candy formulations showed a higher cumulative percentage release (CPR) compared to the control candy formulation (AP00) in all simulated conditions. The CPR of curcumin (180 min) from all emulgel candy formulations was more than two times higher than the control candy across all pH. No significant effect of pH on the curcumin release from the formulations was seen. The CPR from all emulgel candy formulations was higher at pH 5.8 (Figure 9b). AP25C showed the highest CPR of 74.65% at pH 5.8. Hosseini et al. (2022) reported a similar percentage of drug release, where oral jelly candies were designed to replace conventional oral tablets for children [64]. This study reports that polymeric gelling agents can be suitably incorporated into jelly-based formulations for novel dosage forms of important drugs.

**Figure 10:**
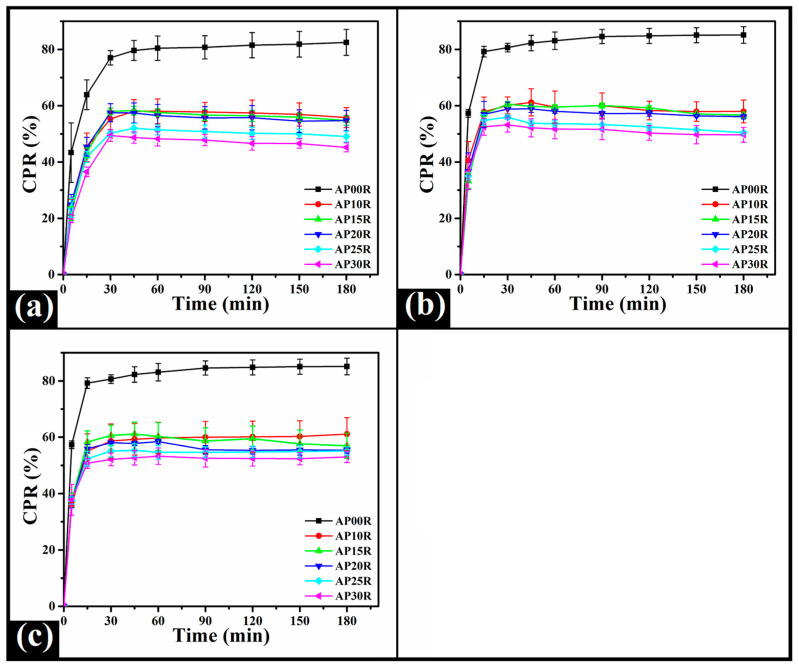
Dissolution profile of the candy formulations containing riboflavin (**a**) CPDR (pH 1.2), (**b**) CPDR (pH 5.8), and (**c**) CPDR (pH 7.2).

Further, the release of riboflavin from AP00 was higher than the emulgel candy formulations. The CPR of riboflavin release from the control formulation was more than 80% at all pHs (Figure 10). A significant decrease in riboflavin was observed in all emulgel candy formulations. The release of riboflavin from the control candy was about 80% within the first 30 min of the study. Such observation indicates the easy impairment of gel stability, enabling the release of riboflavin from the formulations. This burst release of riboflavin from the control formulation could be due to the hydrophilic nature of the riboflavin. Thereafter, the release of riboflavin was nearly constant for the remaining duration of the experiment (3 h). A significantly reduced CPR was observed upon the addition of olive oil within the candy formulations. In other words, there was a decrease in the CPR values with the increase in oil proportion in the candy formulations. This phenomenon was observed in all the simulated gastrointestinal tract conditions. Such a drug release profile suggests a controlled and stable release of the riboflavin molecules from the jelly candy matrix. Moura et al. (2019) investigated the release of anthocyanin from pectin/starch-based jelly candies at pH 1.2 and pH 7.4. The anthocyanin release profile showed that encapsulated samples have a lower release of anthocyanin during the gastric phase of digestion [65].

## 3. Conclusions

The present study formulated a series of emulgel-based jelly candies using sucrose, pectin, and olive oil. Various physicochemical characterizations were performed to evaluate the influence of the olive oil concentration on the properties of the candy formulation. The candy formulations were acidic due to the presence of citric acid. It was observed that the prepared candy formulations had a yellowish tone of the formulations with positive L* and b* values. The FTIR spectroscopy showed that including olive oil decreased the hydrogen bonding intensity within the candy matrices. Thermal analysis revealed the presence of a crystallized sucrose network in the formulations; among all, AP00 possessed the best crystalline structure. The firmness of the candy formulations was lowered with a corresponding increase in the viscous component as olive oil concentration was increased. Disintegration studies suggested that the incorporation of olive oil did not markedly affect the DT at pH 1.2 compared to AP00 (control formulation), except AP15. This suggests the stability of the formulations at an acidic pH that can be useful during the controlled release of the bioactive materials. However, at pHs 5.8 and 7.2, there was a marked reduction in DT as compared to the DT of AP00. Further, it was observed that there was a higher curcumin release from the emulgel candy formulations in comparison to the control candy formulations. However, the release of riboflavin was markedly decreased from the emulgel candy formulation. In short, the preliminary findings of this study suggest that emulgel candy formulations have the potential to carry both hydrophilic and hydrophobic nutraceuticals as a single formulation and may be explored as oral nutraceutical delivery carriers.

## 4. Materials and Methods

### 4.1. Materials

Edible-grade sucrose was purchased from the local grocery store. Pectin (Sweet King India L.L.P, New Delhi, India), citric acid (Central Drug House (P) Ltd., New Delhi, India), olive oil (Ruchi Food Line, Cuttack, Odisha, India), methylparaben (Loba Chemie, Mumbai, India), and propylparaben (Loba Chemie, Mumbai, India) were purchased. Preservative water (PW) was prepared by dissolving methylparaben (C_8_H_8_O_3_; 0.5% *w*/*w*) and propylparaben (C_10_H_12_O_3_; 0.05% *w*/*w*) in distilled water at 60 °C. 

### 4.2. Methodology

#### Preparation of the Candy Formulations

An amount of 5% (*w*/*w*) pectin solution (PS) and 66% *w*/*w* sugar solution were prepared in separate vials. An amount of 5 g of pectin powder was added to PW at 60 °C with continuous stirring at 400 RPM using a temperature-controlled magnetic stirrer (Model RQ-125/D, Remi Motors, India Pvt. Ltd., Mumbai, India). Simultaneously, 40 g of sucrose and 1 g of citric acid were dissolved in distilled water (150 °C) to form a transparent homogeneous solution named sucrose citric acid solution (SCS). A homogenous candy solution was prepared by mixing PS and SCS using the mechanical stirrer at 300 rpm for 3 min. The homogenous solution was then transferred to a container and allowed to solidify at room temperature (25 °C) [31]. The freshly prepared olive oil-free candy formulation was regarded as the control (AP00). 

Emulgel candies were also prepared by following the above procedure with slight modifications. Olive oil was incorporated within PS at various concentrations, which was used for the preparation of the candy formulations. In short, olive oil was first dispersed in PS and homogenized at 1000 rpm for 10 min using a mechanical stirrer (Model RQ-125/D, Remi Motors, India Pvt. Ltd.). Five emulsions were prepared by varying olive oil concentrations, i.e., 10, 15, 20, 25, and 30% *w*/*w* in PS. The oil-in-water (O/W) pectin-based emulsion was further diluted in a water-continuous SCS. Then, the emulgel candies were prepared by following the procedure described above. The prepared candy formulations were denoted as AP10, AP15, AP20, AP25, and AP30, respectively.

Curcumin and riboflavin were used as the model nutraceutical agents. For the samples that contained curcumin, 150 mg of curcumin was added to olive oil while preparing the formulation. Similarly, 250 mg of riboflavin was added to the pectin solution. The nutraceutical-loaded olive oil and PS were used for the development of nutraceutical-loaded emulgel candies. The composition of all the formulations is given in Table 1.

### 4.3. Characterization of the Candies

#### 4.3.1. pH Measurement

The pH of the developed candies was recorded using a digital pH meter (Dolphin instruments, Mumbai, India) at room temperature (25 ± 2 °C) [66]. The measurements were performed in triplicate.

#### 4.3.2. Colorimetry Analysis

The colorimetric parameters of the formulations, L* for lightness (white to black), a* (for red to green), and b* (for blue to yellow),, were analyzed using an in-house developed colorimeter [67]. For this purpose, formulations were prepared in 35 mm Petri dishes, and the colorimetric device captured the image of the formulations [68]. The whiteness index (WI), yellowness index (YI), and absolute color differences (∆E) were calculated using the following equations.

(1) $$WI=100-\sqrt{{\left(100-L^{*}\right)}^{2}+{\left(a^{*}\right)}^{2}+{\left(b^{*}\right)}^{2}}$$

(2) $$YI=142.86\frac{b^{*}}{L^{*}}$$

(3) $$∆E=\sqrt{{\left(L_{C}^{*}-L_{z}^{*}\right)}^{2}+{\left(a_{C}^{*}-a_{z}^{*}\right)}^{2}+{\left(b_{C}^{*}-b_{z}^{*}\right)}^{2}}$$

#### 4.3.3. FTIR Analysis

In order to examine the FTIR spectra of the raw materials and formulations, an FTIR spectrophotometer (AlphaE ATR-FTIR, Bruker, Berlin, Germany) was employed. The spectroscope was working in the Attenuated Total Reflectance (ATR) mode to capture the spectra of the formulations in the wavenumber range of 4000 cm^−1^ to 400 cm^−1^ in frequency. A total of 36 scans were performed for the spectral integration of each formulation [66].

#### 4.3.4. Disintegration Time Study

Using a Tablet Disintegrator Test machine (DT1000, LAB INDIA, Analytical Instruments Pvt. Ltd., Navi Mumbai, India), the amount of time necessary for disintegrating the candy was calculated. In order to disintegrate the candies (2 g), the candies were placed in the sample holder, and 700 mL of the disintegration media was poured into the basket [69]. Then, it was recorded how long it took for each formulation to disintegrate. To simulate the stomach, duodenum, and colon conditions, the dissolving medium’s pH was adjusted as 1.2, 5.8, and 7.2, respectively.

#### 4.3.5. In Vitro Drug Release Study

The release of curcumin and riboflavin from the candy formulations was studied in a tablet dissolution apparatus (DS-8000, Lab India Analytical Instruments Pvt. Ltd., Navi Mumbai, India). In this study, the dissolution apparatus type-I (basket) was employed. The dissolution vessels were filled with 400 mL distilled water containing 0.25% *w*/*v* sodium lauryl sulfate. Candy (~2 g) was placed in the sample holder basket. The dissolution study was performed at 37 ± 0.2 °C for 3 h (Basket rotation speed: 100 RPM). The dissolution study was performed at three different pHs, i.e., simulated gastric (pH 1.2), duodenal (pH 5.8), and intestinal (pH 7.2) conditions. After a specific time (5, 15, 30, 45, 60, 90, 120, 150, and 180 min), 5 mL of the dissolution medium was withdrawn from the dissolution vessel, and the same volume was replaced with the fresh medium. The withdrawn samples were analyzed for curcumin and riboflavin content using a UV-visible spectrophotometer (UV-1900i, Shimadzu, Kyoto, Japan) at 430 nm and 445 nm, respectively [70,71].

#### 4.3.6. Mechanical Analysis

The viscoelastic properties of the candy formulations were investigated by stress relaxation studies using a texture analyzer (Stable Microsystems, TA-HD plus; Godalming, Surrey, UK) [72]. Before starting the analysis, the formulations were cut into 15 cubes and placed on a flat platform. A flat probe having a diameter of 20 mm compressed the formulation to a distance of 2 mm at a test speed of 1 mm s^−1^ and initial triggered force of 5 g. Then the probe was held constant for 60 s, and changes in the force values were recorded. The percentage of the stress relaxation (SR) of the formulation was calculated using Equation (4).

(4) $$\%SR=\left(\frac{F_{0}-F_{60}}{F_{0}}\right)\times 100$$

where F_0_ and F_60_ are the maximum force achieved during compression and the residual force at the end of the relaxation process, respectively.

#### 4.3.7. DSC Analysis

A differential scanning calorimeter (DSC 200F3 Maia, Netzsch, Selb, Germany) was used to analyze the thermal behavior of the candy formulations. The sample was accurately weighed (~5 mg) and sealed in an aluminum pan with pierced lid. An empty aluminum pan with a pierced lid was taken as a reference. The formulations were analyzed in a temperature range between 0 °C and 300 °C at a scanning rate of 10 °C/min [64].

#### 4.3.8. Statistical Analysis

The findings of each experiment were carried out in triplicate, and they are presented as mean ± standard deviation (SD). Utilizing IBM SPSS Statistics 20 software (Inc., Chicago, IL, USA), statistical analysis was carried out. With a significance threshold of *p* < 0.05, a one-way analysis of variance (ANOVA) and Tukey post hoc test were used to identify statistically significant values.

## Figures and Tables

**Table 1:** Composition of the emulgel candies.

S. No.	Sample Name	SCS (g)	Emulsion		Nutraceutical Agents	
			PS (g)	Olive Oil (g)	Curcumin (mg)	Riboflavin (mg)
1	AP00	35	15	--	--	--
2	AP10	35	13.5	1.5	--	--
3	AP15	35	12.75	2.25	--	--
4	AP20	35	12	3	--	--
5	AP25	35	11.25	3.75	--	--
6	AP30	35	10.5	4.5	--	--
7	AP00-C	35	15	--	150	--
8	AP10-C	35	13.5	1.5	150	--
9	AP15-C	35	12.75	2.25	150	--
10	AP20-C	35	12	3	150	--
11	AP25-C	35	11.25	3.75	150	--
12	AP30-C	35	10.5	4.5	150	--
13	AP00-R	35	15	--	--	250
14	AP10-R	35	13.5	1.5	--	250
15	AP15-R	35	12.75	2.25	--	250
16	AP20-R	35	12	3	--	250
17	AP25-R	35	11.25	3.75	--	250
18	AP30-R	35	10.5	4.5	--	250

## Data Availability

The data presented in this study are available on request from the corresponding author.

## Supplementary Materials

The following supporting information can be downloaded at: https://www.mdpi.com/article/10.3390/gels9060466/s1, Figure S1: pH of emulsion-filled candy formulations. Columns having the same alphabet suggests that they are statistically similar by Tukey HSD test with *p* > 0.05.; Table S1: Absorption signal at different wavenumbers.
