# Supplementary material for: Formulation and Characterization of Emulgel-Based Jelly Candy: A Preliminary Study on Nutraceutical Delivery
Source: Gels. 2023 Jun 6;9(6):466. doi: 10.3390/gels9060466 (PMC10298008; doi:10.3390/gels9060466)
Supplement: Supplementary file 1 [file gels-09-00466-s001.zip › gels-2381266-supplementary.pdf]

Formulation and Characterization of Emulgel-Based Jelly Candy: A Preliminary Study on Nutraceutical Delivery

Somali Dhal [1,†], Anupam Pal [2,†], Anna Gramza-Michalowska [3], Doman Kim [4], Biswaranjan Mohanty [2,*], Sai S. Sagiri [5] and Kunal Pal [1,*]

Supplementary data

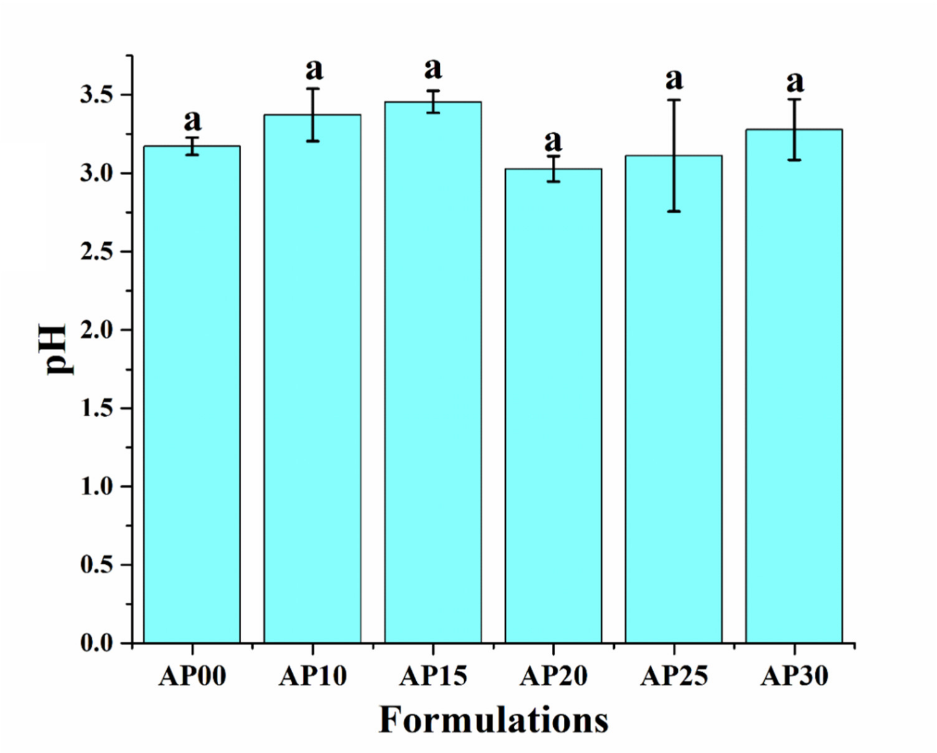

Figure S1: pH of emulsion-filled candy formulations. Columns having the same alphabet suggests that they are statistically similar by Tukey HSD test with $p > 0.05$.

Table S1. Absorption signal at different wavenumbers.

**Wavenumber (cm^{-1})**	**Functional group**	**References**
3277	OH stretching	(Patil and Netravali 2019)
2926	Asymmetric C-H stretching of aliphatic groups of the sucrose molecule.	(Tohamy et al. 2022)
1642	Stretching of OH, COO− (asymmetric), COO− (symmetric), and C O C, respectively	(Romano et al. 2016)
1414	Torsion OH, twisting H_2O	(Brizuela et al. 2014)
1345	Symmetric scissoring of the C-OH	(Brizuela et al. 2014)
1022	Stretching of C–C	(Bichara et al. 2014)
